# Remimazolam versus propofol for postoperative ICU sedation in mechanically ventilated cancer patients: a noninferiority randomized clinical trial

**DOI:** 10.3389/fphar.2026.1784928

**Published:** 2026-03-31

**Authors:** Congcong Zhao, Xiaoxuan Fan, Leying Li, Zihan Nan, Xin lin, Yaqi Han, Yanshuo Wu, Yanling Yin, Yan Xin, Zhenjie Hu

**Affiliations:** 1 Department of Intensive Care Unit, The Fourth Hospital of Hebei Medical University, Shijiazhuang, China; 2 Postgraduate of Intensive Care Unit, The Fourth Hospital of Hebei Medical University, Shijiazhuang, Hebei, China; 3 Department of Intensive Care Unit, The Third Hospital of Shijiazhuang, Shijiazhuang, China; 4 Hebei Key Laboratory of Critical Disease Mechanism and Intervention, The Fourth Hospital of Hebei Medical University, Shijiazhuang, Hebei, China

**Keywords:** cancer, mechanical ventilation, propofol, remimazolam, sedation

## Abstract

**Objective:**

This study aimed to compare the efficacy and safety of the novel ultra-short-acting benzodiazepine, remimazolam, versus propofol for short-term sedation in postoperative cancer patients during mechanical ventilation in the ICU.

**Methods:**

This single-center, randomized noninferiority clinical trial was conducted from February 1st to 31 August 2024. Adult postoperative cancer patients requiring mechanical ventilation in the ICU were randomized to receive either remimazolam or propofol. The primary outcomes were the time to achieve target sedation (Richmond Agitation-Sedation Scale, RASS, −2 to 0) and the percentage of time within the target range without rescue sedation.

**Results:**

A total of 80 patients were enrolled and randomly assigned to the remimazolam group (n = 40) or the propofol group (n = 40). The time to achieve target sedation was comparable between the remimazolam and propofol groups [median 3.0 (IQR 2.0–4.0) vs. 3.0 (IQR 2.0–4.5) minutes, P = 0.590]. The median percentage of time within the target sedation range was also similar (80.0% vs. 73.2%, P = 0.546). However, remimazolam demonstrated a superior safety profile, with significantly lower incidences of hypotension (15.0% vs. 42.5%, P = 0.007) and respiratory depression (5.0% vs. 20.0%, P = 0.043). No significant differences were observed in secondary outcomes, including duration of mechanical ventilation, ICU length of stay, mortality, or costs.

**Conclusion:**

In mechanically ventilated, postoperative cancer patients, remimazolam provides non-inferior sedation efficacy compared to propofol, but with a significantly more favorable safety profile, particularly regarding hemodynamic and respiratory stability. These findings position Remimazolam as a promising and safer alternative for sedation in this vulnerable population.

## 1 Introduction

Postoperative admission to the intensive care unit (ICU) is common for cancer patients, and the number of ICU admission is increasing (Azoulay et al., 2017; Zampieri et al., 2021). Most of these patients require mechanical ventilation in order to prevent major cardiovascular and pulmonary complications. ICU environments induce significant physiological stress, necessitating substantial analgesic and sedative support (Devlin et al., 2018). Sedatives are routinely administered to enhance comfort, alleviate anxiety, and mitigate ventilator-related distress (Barr et al., 2013). Current sedatives such as midazolam, propofol, and dexmedetomidine each carry clinical limitations (Choi et al., 2018; Forman, 2011; Kim et al., 2015; Montravers et al., 1994). The ideal sedative should feature rapid onset, predictable offset, minimal adverse effects, organ-independent metabolism, and availability of an antagonist.

Remimazolam is a novel ultra-short-acting benzodiazepine derivative characterized by rapid onset and offset (Sneyd et al., 2021). Unlike midazolam, its ester-based side chain enables hydrolysis by nonspecific tissue esterases into an inactive carboxylic acid metabolite. This organ-independent metabolic pathway minimizes accumulation risk during prolonged infusion (Freyer et al., 2019; Wesol et al., 2016; Zhou et al., 2017). Critically, Remimazolam demonstrates superior hemodynamic stability. A recent RCT in interventional neuroradiology reported significantly lower hypotension rates and faster recovery with Remimazolam versus propofol (Lee et al., 2024). Furthermore, Remimazolam’s effects can be reversed by flumazenil, a benzodiazepine antagonist, enabling rapid restoration of consciousness and potentially reducing delirium risk (Chen et al., 2020). These pharmacological advantages position Remimazolam as a promising ICU sedative.

Although Remimazolam has demonstrated advantage in procedural sedation and short-term surgical anesthesia (Sneyd et al., 2021; Tang et al., 2022a), but few studies focus on its use in postoperative ICU patients. A phase I study in non-cardiac post-surgical patients identified an optimal maintenance dose (0.125–0.15 mg/kg/h) for light-to-moderate sedation (Tang et al., 2022a). A subsequent pilot study compared Remimazolam with propofol for long-term sedation (>24 h) in 60 mechanically ventilated ICU patients, reporting comparable sedation efficacy, similar adverse event rates, and no significant differences in ventilator-free days or mortality (Tang et al., 2022b). Despite promising preclinical and early clinical data, robust evidence supporting Remimazolam’s ICU application remains scarce. The efficacy and safety profile of Remimazolam specifically in postoperative cancer patients is yet to be established.

Therefore, this study aimed to evaluate the effectiveness and safety of Remimazolam versus propofol for maintaining mild sedation in mechanically ventilated patients following cancer surgery in the ICU.

## 2 Methods

We conducted a single-center, prospective, randomized, single-blind, noninferiority clinical trial in the ICU of the Fourth Hospital of Hebei Medical University from February 1st to 31 August 2024. Ethical approval from the local institutional ethics committee (Approval No. 2023096), and all the patients signed informed consents. This study was prospectively registered at ClinicalTrials.gov (ChiCTR2400080234) prior to participant enrollment.

## 3 Patients

Adult patients (≥18 years) admitted to the ICU after elective cancer resection surgery and requiring light sedation (target Richmond Agitation-Sedation Scale (RASS)–2 to 0) during postoperative mechanical ventilation were eligible for inclusion.

Patients were excluded if they:

1. Required deep sedation anticipated on the basis of predefined clinical criteria;
2. Had chronic use of benzodiazepines or other psychotropic agents for >3 months prior to surgery;
3. Had a documented history of substance or alcohol abuse within the past year based on medical records or clinician assessment;
4. Were pregnant or lactating;
5. Had known hypersensitivity to remimazolam, propofol, or formulation components;
6. Were participating in another interventional drug trial or lacked the capacity to provide informed consent without a legally authorized representative.

## 4 Randomization and blinding methods

Eligible patients were randomized in a 1:1 ratio to the remimazolam group or the propofol group. The randomization sequence was computer-generated by an independent biostatistician who was not involved in patient recruitment, clinical management, or data collection. Because the two sedatives differ in appearance and preparation, blinding of patients and clinical staff was not feasible. Therefore, a single-blind design was adopted. Outcome assessors and data analysts remained blinded to treatment allocation throughout the study, and all data were coded before statistical analysis to prevent inadvertent unblinding.

## 5 Analgesia and sedation

Upon enrollment, all ongoing sedatives and analgesics were discontinued. Analgesia was maintained with remifentanil (Yichang Humanwell Pharmaceutical Co., Ltd., China) at 1.2–6.0 μg/kg/h, titrated to a Critical-Care Pain Observation Tool (CPOT) score of 0, with close monitoring of hemodynamics and adverse effects.

Before randomization, propofol (0.3–4.0 mg/kg/h) was permitted if necessary to achieve light sedation (RASS –2 to 0). After randomization, the assigned study drug was administered: Remimazolam Tosilate (Hengrui Pharmaceutical Co., Ltd., China) 0.1 mg/kg loading, 0.1–0.3 mg/kg/h maintenance; Propofol (Sichuan Guorui Pharmaceutical Co., Ltd., China) 2.0 mg/kg loading, 0.5–4.0 mg/kg/h maintenance.

RASS was assessed every 4 h, and infusion rates were adjusted accordingly. Rescue boluses were allowed for agitation (RASS >0; remimazolam 0.05–0.1 mg/kg or propofol 0.3–0.5 mg/kg), and study drug was reduced or withheld if oversedated (RASS <–2). If maximum doses failed, adjunctive dexmedetomidine (0.2–0.7 μg/kg/h; National Pharmaceutical Industry Co., Ltd., China) was permitted.

Sedation was discontinued upon extubation, ICU discharge, planned interruption >12 h, or 5 days post-randomization.

## 6 Outcomes

### 6.1 Primary outcomes

Time to achieve target sedation: Defined as the time interval (in minutes) from the initiation of the study drug infusion to the first documented Richmond Agitation-Sedation Scale (RASS) score between −2 and 0.

Percentage of time within target sedation range: Calculated as the proportion of all scheduled RASS assessments (performed every 4 h) during the study period in which the patient’s RASS score was maintained between −2 and 0, excluding any assessments following the administration of rescue sedation (i.e., dexmedetomidine).

### 6.2 Secondary outcomes

Including the duration of mechanical ventilation, ventilator-free hours during ICU stay, ICU length of stay, ICU mortality, and 28-day mortality.

### 6.3 Incidence of adverse events

Including Respiratory depression, hypotension, bradycardia, Hepatic injury, Renal injury, and Gastrointestinal events. The specific definitions are provided in Additional file 1.

### 6.4 Economic analysis

Direct medication costs were compared between groups, including total sedative and analgesic costs. All costs are presented in Chinese Yuan (CNY) and include only drug acquisition costs, excluding other ICU-related costs.

## 7 Statistical analysis

The sample size calculation was based on a non-inferiority design with a margin of −0.1. Utilizing preliminary data indicating mean sedation times of 2.8 (±0.7) minutes for propofol and 3.2 (±0.8) minutes for remimazolam, we determined that 36 participants per group would provide 80% power at a one-sided α level of 0.025. To account for a potential 10% dropout rate, the final sample size was 40 participants per group.

Statistical analyses were performed using SPSS (version 26.0; IBM Corp., Armonk, NY, United States). For data with a normal distribution, continuous variables were presented as the mean ± standard deviation (SD), whereas non-normally distributed continuous variables were reported as the median [interquartile range (IQR)]. Between-group comparisons were conducted using independent samples t-tests or Mann-Whitney U tests, as appropriate. Categorical variables were expressed as frequencies (%) and compared using Chi-square test or Fisher’s exact test. Missing data were not imputed, as the amount of missingness was minimal. Statistical significance was defined as a two-tailed P value <0.05.

## 8 Results

**FIGURE 1:**
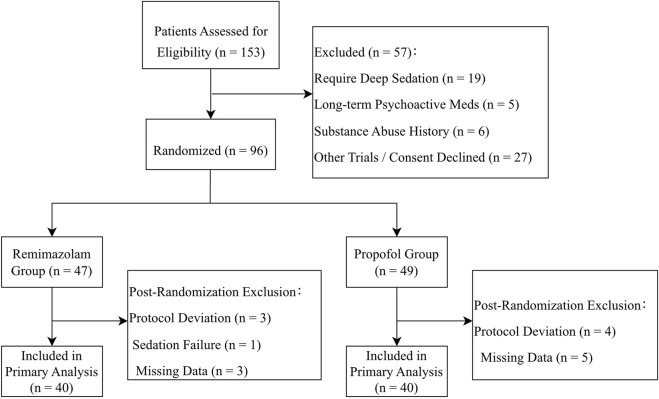
Study protocol flowchart.

A total of 153 patients were screened for eligibility, with 80 meeting inclusion criteria and subsequently enrolled. Participants were randomized to receive either remimazolam (n = 40) or propofol (n = 40). All enrolled patients received protocol-directed sedation and underwent continuous monitoring in the ICU. The participant flow diagram is presented in Figure 1. The cohort had a median age of 68 years, with 62.5% being male. Colorectal cancer represented the most prevalent malignancy. No significant differences in baseline characteristics were observed between the two groups (Table 1).

**TABLE 1:** Baseline characteristics of the patients.

​	Remimazolam (n = 40)	Propofol (n = 40)	*P* Value
Age, years, median [IQR]	69 (61–75)	69 (60–76)	0.881
Male, n (%)	27 (67.5)	23 (57.5)	0.356
Weight, kg, mean ± SD	61.8 ± 11.0	62.7 ± 10.2	0.702
Height, cm, mean ± SD	166.8 ± 8.5	164.0 ± 8.1	0.146
BMI, kg/m^2^, mean ± SD	22.1 ± 3.0	23.3 ± 3.1	0.109
HR, bpm, median [IQR]	81 (69–93)	87 (78–92)	0.209
MAP, mmHg, median [IQR]	98 (85–105)	94 (79–110)	0.881
CVP, cmH_2_O, mean ± SD	8.1 ± 3.1	8.2 ± 3.0	0.807
Use of vasoactive drug, n (%)	6 (15.0)	5 (12.5)	0.745
Apache-II score, median [IQR]	12.0 (11.0–15.0)	12.0 (11.0–14.5)	0.839
SOFA score, median [IQR]	4 (2–6)	4 (2–5)	0.446
CPOT score, median [IQR]	0 (0–0)	0 (0–0)	0.155
RASS score, median [IQR]	−1 (-2-0)	−1 (-2-0)	0.163
Previous medical history, n (%)			
Hypertension	22 (55.0)	20 (50.0)	0.654
Diabetes mellitus	5 (12.5)	12 (30.0)	0.056
Coronary heart disease	9 (22.5)	9 (22.5)	1.000
COPD	3 (7.5)	6 (15.0)	0.241
Others	28 (70.0)	26 (65.0)	0.633
Surgical etiology, n (%)			
Colorectal cancer	21 (52.5)	18 (45.0)	0.655
Gynecological tumor	5 (12.5)	7 (17.5)	0.755
Esophageal cancer	5 (12.5)	3 (7.5)	0.712
Lung cancer	3 (7.5)	2 (5.0)	1.000
Gastric cancer	2 (5.0)	3 (7.5)	1.000
Others	7 (17.5)	4 (10.0)	0.518

APACHE II, Acute Physiology and Chronic Health Evaluation II; BMI, body mass index; CPOT, critical care pain observation tool; COPD, chronic obstructive pulmonary disease; CVP, central venous pressure; HR, heart rate; MAP, mean arterial pressure; RASS, Richmond Agitation-Sedation Scale; SOFA,sequential organ failure assessment.

### 8.1 Primary outcome

**FIGURE 2:**
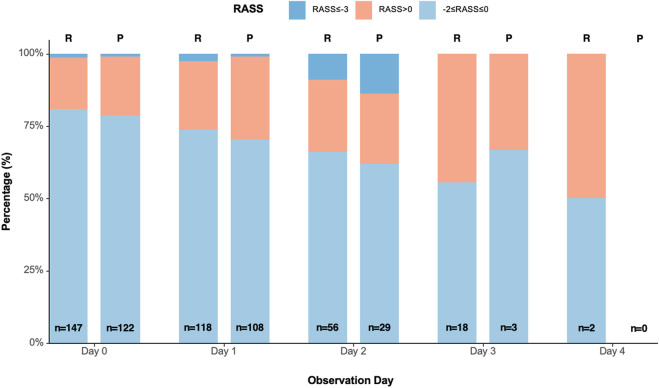
Precentage of RASS scores in the remimazolam and propofol groups. Distribution of RASS scores ranged from −5 (unarousable) to +4 (combative), with a score between −2 and 0 representing the target sedation range. RASS, Richmond Agitation-Sedation Scale; R, Remimazolam group; P, Propofol group.

The median time to achieve target sedation was 3.0 (2.0–4.0) minutes in the remimazolam group and 3.0 (2.0–4.5) minutes in the propofol group (P = 0.590; Table 2). During the observation period, 341 and 262 RASS assessments were recorded in the Remimazolam Tosilate and propofol groups, respectively. The distribution of sedation depth is presented in Figure 2. The median percentage of time in the target sedation range without rescue sedation was 80.0% (64.4%–83.3%) in the remimazolam group and 73.2% (66.7%–83.3%) in the propofol group (P = 0.546, Table 2). Rescue sedation was administered to 5 (12.5%) patients in the remimazolam group and 4 (10.0%) in the propofol group, with no statistically significant difference observed between groups (Table 2).

**TABLE 2:** Outcomes between the propofol and remimazolam tosilate groups.

​	Remimazolam (n = 40)	Propofol (n = 40)	*P* Value
Time to achieve target sedation, min, median [IQR]	3.0 (2.0–4.0)	3.0 (2.0–4.5)	0.590
Percentage of target sedation achievement, median [IQR]	80.0% (64.4%–83.3%)	73.2% (66.7%–83.3%)	0.546
Ventilator time in ICU, h, median [IQR]	23.0 (19.0–41.3)	20.5 (13.0–32.5)	0.061
Free-ventilator time in ICU, h, median [IQR]	47.5 (32.8–70.8)	65.5 (46.0–95.0)	0.066
ICU length of stay, d, median [IQR]	5.0 (4.0–6.0)	4.5 (4.0–6.0)	0.504
ICU mortality, n (%)	0	0	​
28-day mortality, n (%)	1 (2.5)	2 (5.0)	1.000
Dose of study drug, mg/kg/h,median [IQR]	0.104 (0.101–0.115)	0.537 (0.509–0.598)	<0.001*
Dose of remifentanil, μg/kg/h,median [IQR]	3.7 (3.2–5.4)	3.2 (2.6–5.1)	0.312
Rescue sedation^#^, n (%)	5 (12.5)	4 (10.0)	1.000

d, days; h,hours; min, minutes. #Rescue sedation was defined as the administration of dexmedetomidine for salvage sedation. *P < 0.05.

### 8.2 Secondary outcomes

Secondary outcomes are presented in Table 2. The median duration of mechanical ventilation was 23.0 (19.0–41.3) hours in the remimazolam group versus 20.5 (13.0–32.5) hours in the propofol group (P = 0.061). Ventilator-free hours during ICU stay did not differ significantly between groups. Furthermore, no significant differences were observed in ICU length of stay, ICU mortality, or 28-day mortality.

### 8.3 Adverse events

**FIGURE 3:**
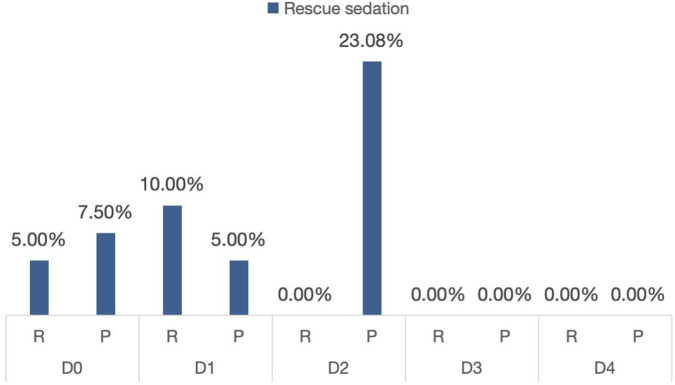
Incidence of adverse reactions in the propofol and remimazolam groups. Incidence of adverse events requiring rescue sedation on postoperative days 0–4 (D0-D4). *P < 0.05 for between-group comparison. R, Remimazolam group; P, Propofol group.

Adverse events occurred in 22 (55%) remimazolam-treated patients and 32 (80%) propofol-treated patients. While overall adverse event rates did not differ significantly between groups (Figure 3), the remimazolam group demonstrated significantly lower incidences of specific complications. Compared with propofol group, remimazolam group occurred lower respiratory depression [8 (5.0%) propofol-treated patients vs. 2 (20.0%) remimazolam-treated patients, P = 0.043] and hypotension [17 (42.5%) propofol-treated patients vs. 6 (15.0%) remimazolam -treated patients, P = 0.007].

### 8.4 Economic indicators

The median sedation maintenance dose was 0.104 mg/kg/h for remimazolam versus 0.537 mg/kg/h for propofol. Despite this dose differential, sedation medication costs showed no statistically significant difference between groups (281.0 ¥ vs. 396.2 ¥; P = 0.068). Similarly, other economic indicators, including analgesics costs,ICU-related costs, and total hospitalization costs demonstrated comparable values without statistical significance (Table 3).

**TABLE 3:** Economic indicators between the propofol and remimazolam tosilate groups.

​	Remimazolam	Propofol	*P* Value
Sedatives expenses, yuan, median [IQR]	281.0 (168.6–505.7)	396.2 (198.1–417.7)	0.068
Analgesics expenses, yuan, median [IQR]	719.2 (359.6–1,078.8)	719.2 (359.6–719.2)	0.690
ICU expenses, yuan, median [IQR]	18,932.3 (14,740.4–29323.1)	21,871.0 (16,309.8–30058.8)	0.424
Hospitalization expenses, yuan, median [IQR]	70,444.3 (58,622.0–83029.2)	82,339.5 (65,950.8–92158.2)	0.057

Economic data are presented as median (interquartile range, IQR). ICU, intensive care unit.

Additional file1. Incidence of adverse reactions in the propofol and Remimazolam Tosilate groups.

Data are presented as n (%). * indicates a statistically significant difference (P < 0.05) between the Propofol and Remimazolam groups.

## 9 Discussion

This is the first study to compare the sedative efficacy, safety, and economic characteristics of remimazolam tosilate and propofol in mechanically ventilated patients after tumor surgery. The findings demonstrate comparable sedation efficacy and economic profiles between the two agents. However, remimazolam was associated with significantly lower incidences of hypotension and respiratory depression, suggesting a potential safety advantage. These results provide novel clinical evidence to guide sedative selection for critically ill postoperative cancer patients.

Continuous sedation is often required in ICU postoperative patients to mitigate stress responses, enhance comfort, and improve treatment compliance. Nevertheless, sedation management faces challenges including significant interindividual variability and substantial drug-related adverse effects (Bevans et al., 2017; Boncyk et al., 2024; Northam and Phillips, 2024). As a novel short-acting benzodiazepine, remimazolam features rapid onset, brief duration of action, and reversibility (Doi et al., 2020; LI et al., 2024; Stöhr et al., 2021). Its application in perioperative sedation has gradually attracted attention.

Propofol, as a widely used sedative in critical care settings, has well-established efficacy (Brohan and Goudra, 2017; Walsh, 2018). The results of this study indicate that Remimazolam Tosilate achieves non-inferior short-term sedative efficacy compared to propofol in mechanically ventilated postoperative cancer patients in the ICU, providing stable and target-consistent sedation levels. Consistent with our findings, Zhang et al. reported comparable onset times and maintenance of target sedation depth between remimazolam and propofol in a randomized trial of patients undergoing gastrointestinal endoscopy (Zhang et al., 2022). Wu et al.'s prospective study in ICU patients also confirmed that remimazolam delivers predictable sedation while significantly reducing risks of oversedation and delayed recovery (Wu et al., 2023). Moreover, no significant differences were observed between the agents regarding duration of mechanical ventilation, ICU length of stay, or mortality. Our data provide new clinical evidence supporting remimazolam tosilate’s application for postoperative cancer patients, particularly those requiring light sedation in the ICU setting.

In terms of safety, our study found that remimazolam was significantly superior to propofol in reducing the incidence of respiratory depression and hypotension. Similarly, a recent meta-analysis showed that remimazolam was apparently associated with fewer adverse drug events in comparison to propofol for general anesthesia in patients undergoing surgery (Huang et al., 2024). Existing studies have shown that propofol induces dose-dependent hypotension by dilating peripheral blood vessels, which may precipitate circulatory collapse in severe cases (Guo et al., 2022; Lewis et al., 2022). Furthermore, its central respiratory depressant effects frequently reduce tidal volume and cause hypoventilation (Lewis et al., 2022). In contrast, remimazolam may be particularly suitable for patients with hemodynamic instability or heightened susceptibility to respiratory depression, such as the elderly, patients with cardiovascular diseases, or those with brain injuries. This pharmacological profile enhances sedation controllability and safety.

We found no difference in hospitalization costs between the remimazolam group and propofol groups. However, the cost of sedative drugs in the remimazolam group was slightly lower. This trend is consistent with the findings of Yao et al. (2023). The reduced incidence of complications such as respiratory depression and hypotension with remimazolam may decrease the need for vasoactive agents and additional ventilatory support, potentially conferring indirect economic advantages. The non-significant econometric results in our study may be attributable to the limited sample size, population specificity, and substantial ICU expenditures. Further investigations are warranted to identify patient populations most likely to derive economic benefit from Remimazolam Tosilate sedation.

This randomized controlled trial comprehensively evaluated the clinical potential of remimazolam tosilate in mechanically ventilated patients after tumor surgery from multiple aspects, including sedative efficacy, safety, and economic indicators. However, several limitations warrant consideration. First, as a single-center pilot study with a relatively small sample size, the evidence level of the research results is low and require validation in larger cohorts. Second, the exclusive focus on post-surgical cancer patients limits generalizability to broader critically ill populations. Future research should prioritize evaluating remimazolam’s efficacy and safety in patients with distinct pathophysiological states. Additionally, this study did not evaluate long-term safety outcomes, including post-discontinuation cognitive recovery, delirium incidence, and long-term survival.

## 10 Conclusion

In conclusion, this study demonstrates that remimazolam tosilate provides non-inferior short-term sedation efficacy compared with propofol in mechanically ventilated postoperative cancer patients in the ICU. Crucially, remimazolam tosilate is associated with significantly lower incidences of respiratory depression and hypotension. These findings position remimazolam as a safer alternative for target sedation management in high-risk surgical populations.

## Data Availability

The raw data supporting the conclusions of this article will be made available by the authors, without undue reservation.
